# Loss of mtDNA activates astrocytes and leads to spongiotic encephalopathy

**DOI:** 10.1038/s41467-017-01859-9

**Published:** 2018-01-04

**Authors:** Olesia Ignatenko, Dmitri Chilov, Ilse Paetau, Elena de Miguel, Christopher B. Jackson, Gabrielle Capin, Anders Paetau, Mugen Terzioglu, Liliya Euro, Anu Suomalainen

**Affiliations:** 1 Research Programs Unit, Molecular Neurology, Biomedicum Helsinki, Haartmaninkatu 8, University of Helsinki, Helsinki, 00014 Finland; 2 Department of Pharmacology, Faculty of Medicine, Haartmaninkatu 8, University of Helsinki, Helsinki, 00014 Finland; 3 Department of Pathology, Huslab and Helsinki University Hospital and Medicum, Haartmaninkatu 3, University of Helsinki, Helsinki, 01051 Finland; 4 Neuroscience Center, Viikinkaari 4, University of Helsinki, Helsinki, 00014 Finland; 5 Department of Neurosciences, Haartmaninkatu 4, Helsinki University Hospital, Helsinki, 01051 Finland

## Abstract

Mitochondrial dysfunction manifests as different neurological diseases, but the mechanisms underlying the clinical variability remain poorly understood. To clarify whether different brain cells have differential sensitivity to mitochondrial dysfunction, we induced mitochondrial DNA (mtDNA) depletion in either neurons or astrocytes of mice, by inactivating Twinkle (TwKO), the replicative mtDNA helicase. Here we show that astrocytes, the most abundant cerebral cell type, are chronically activated upon mtDNA loss, leading to early-onset spongiotic degeneration of brain parenchyma, microgliosis and secondary neurodegeneration. Neuronal mtDNA loss does not, however, cause symptoms until 8 months of age. Findings in astrocyte-TwKO mimic neuropathology of Alpers syndrome, infantile-onset mitochondrial spongiotic encephalopathy caused by mtDNA maintenance defects. Our evidence indicates that (1) astrocytes are dependent on mtDNA integrity; (2) mitochondrial metabolism contributes to their activation; (3) chronic astrocyte activation has devastating consequences, underlying spongiotic encephalopathy; and that (4) astrocytes are a potential target for interventions.

## Abstract (web summary)

Astrocytes in the brain are metabolically dynamic. Here, Ignatenko, Chilov and colleagues delete mitochondrial DNA (mtDNA) in a cell type specific manner, and show that inactivation of mtDNA helicase Twinkle in astrocytes leads to spongiotic encephalopathy.

## Introduction

Primary mitochondrial dysfunction is the most common cause of inherited metabolic disease^1^. The central nervous system manifestations of mitochondrial diseases are typically progressive, with manifestations ranging from infantile catastrophic epileptic encephalopathy to ataxia-epilepsy syndrome, leukoencephalopathy, neurodegeneration or parkinsonism^1,2^. Among the most severe disorders are Leigh syndrome, a subacute necrotizing encephalopathy, and Alpers–Huttenlocher syndrome with spongiotic changes, laminar cortical necrosis and liver failure^3–5^. These devastating, lethal disorders were both characterized originally by their typical neuropathological signs, which are the key determinant of the clinical diagnosis even today^6,7^.

Alpers–Huttenlocher syndrome is caused by recessive mutations in mitochondrial DNA (mtDNA) maintenance proteins: the nuclear-encoded DNA polymerase gamma or replicative helicase Twinkle^8,9^. These proteins, together with the mitochondrial transcription factor A (TFAM) form the minimal mtDNA replisome, and are essential for faithful mtDNA replication^10^: their total inactivation in mice is embryonic lethal at ~E8.5^11–13^. Knocking out TFAM or expressing mitochondrial-targeted endonuclease in forebrain or dopaminergic neurons of mice resulted in progressive loss of mtDNA, eventual death of the targeted neurons and shortened lifespan of the animals^14–16^. The direct consequence of mtDNA loss is deficiency of the 13 mtDNA-encoded subunits that together with 76 nuclear-encoded subunits build up the multisubunit enzyme complexes of oxidative phosphorylation (OXPHOS) in which the Complexes I–IV (CI-CIV) form the respiratory chain (RC) and Complex V is the ATP synthase. The late manifestation of neurological symptoms in neuronal TFAM-knockout mice and the mitochondrial endonuclease over-expressor mice indicates that neurons can sustain relatively prolonged periods of mtDNA depletion and severe RC deficiency, questioning the role of neurons as the primary culprit in mtDNA maintenance disorders.

Astrocytes are the most abundant cell type in the brain, providing trophic and metabolic support for neurons, pruning synapses and synthesizing neurotransmitters^17^. They are metabolically dynamic, and considered to be glycolytic, i.e. to have capacity to perform non-oxygen-dependent ATP-synthesis and survive OXPHOS deficiency in vivo^18,19^. The contribution of astrocytes in mitochondrial disorders has been considered to be secondary, forming a glial scar at the sites of neuronal damage. However, no evidence exists of the importance of mtDNA maintenance in astrocytes in vivo.

Here we investigate the consequences of inactivation of Twinkle, the mtDNA replicative helicase, in astrocytes or neurons in vivo. We show that mtDNA maintenance is critical for both cell types, but in a different manner. Progressive loss of Twinkle and mtDNA in neurons causes late-onset neurodegeneration and rapid progressive late-stage neurological deterioration. Twinkle-KO in astrocytes, however, leads to wide-spread activation of the cell type, severe early-onset progressive neurological disease, accompanied by spongiotic encephalopathy and inflammation. The results propose that primary astrocyte dysfunction underlies mitochondrial spongiotic encephalopathies, such as Alpers syndrome, caused by mtDNA maintenance defects.

## Results

### Generation of constitutive and conditional *Twinkle*-KO mice

**Fig. 1:**
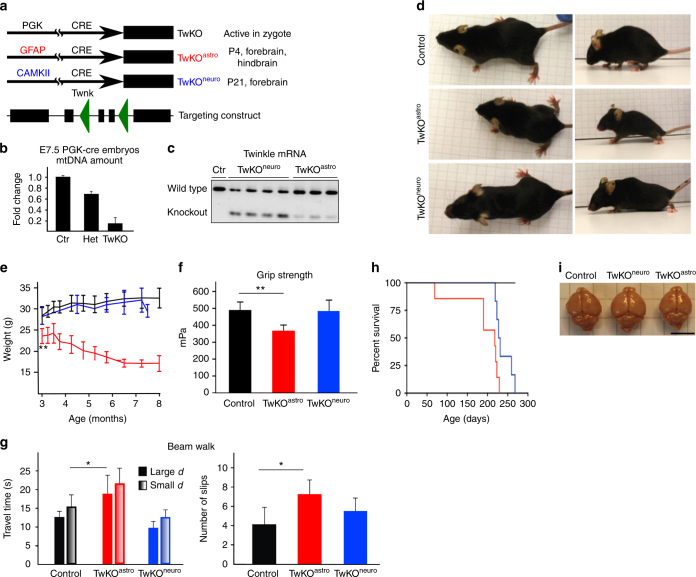
Knockout of mtDNA maintenance in astrocytes causes early-onset neurodegenerative disease. **a** *Cre-*promoters and timing of activation (PGK = phosphoglycerate kinase, GFAP = glial fibrillary-acidic-protein, CaMKII = calcium/calmodulin-dependent protein kinase-II). Targeting construct: Boxes = exons, triangles = LoxP sites. **b** MtDNA amount in constitutive Twinkle knockout (TwKO), embryonal day 7.5 (Ctr = control, Het = heterozygote; TwKO = homozygous). **c** Size of Twinkle-cDNA in TwKO lines; cerebral cortex. **d** TwKO mouse phenotypes at the age of 6 months. TwKO^astro^ mice: small, paraparetic posture. TwKO^neuro^ mice are control-like. **e** Body weight progression: red = TwKO^astro^, blue = TwKO^neuro^, black = Control. **p < 0.01 for the first time-point; subsequent time-points *p* < 0.05. **f** Automated grip strength test for forelimbs (6 months of age). **g** Beam walk test for motor coordination (large *d* = large diameter, small *d* = small diameter beam). **h** Kaplan–Meier survival curves. Red = TwKO^astro^, blue = TwKO^neuro^, black = Control. **i** Brains of TwKO^astro^, TwKO^neuro^, and Control mice are similar. Scale bar is 0.5 cm. The data are presented as mean and error bars indicate standard deviation. Statistical significance: *p < 0.05, **p < 0.01

To clarify whether mtDNA maintenance is similarly essential for neurons and glia, we designed cell-type-specific conditional knockout mice for the *Twnk* gene encoding mitochondrial DNA helicase Twinkle (TwKO) (Fig. 1a). We first generated early ubiquitous inactivation of Twinkle, using PGK-promoter, driving cre-recombinase expression from the zygote stage^20^. These homozygous full-TwKO embryos showed drastically reduced mtDNA content already at E7.5 leading to lethality at E8.5, as previously described^12^ (Fig. 1b). Then we targeted *Twinkle* knockout (TwKO) to postnatal forebrain neurons (TwKO^neuro^; CamKII-cre) or astrocytes (TwKO^astro^; mouseGFAP73.12-cre) (Fig. 1a). From the existing transgenic cre-lines targeting astrocytes, we chose the mGFAP73.12 promoter since (1) it starts to express postnatally, allowing brain to develop normally; (2) embryonic neuronal stem cells remain unaffected; (3) the profile of expression is well characterized and specific for astrocytes^21–23^, except for some expression in a population of adult neuronal stem cells generating a small number of neurons during adult life. Furthermore, cre-activation affects Twinkle protein and mtDNA amounts with a delay, the latter having a half-life in brain of several weeks^24^. Reporter mice expressing GFP upon recombination, crossed with mGfap73.12, indeed did not demonstrate GFP-expression at P2, and in adult mice showed intensive expression in cortex, hippocampus and cerebellum (Supplementary Fig. 1), and low expression in midbrain, resembling the pattern of expression previously reported for this promoter. The TwKO^neuro^ and TwKO^astro^ lines were viable, and the *cre* transgene segregated to Mendelian ratio (TwKO^neuro^ 42/78 and TwKO^astro^ 24/41). The successful TwKO was confirmed by *Twinkle* cDNA analysis (Fig. 1c).

### TwKO in astrocytes causes early-onset neurological disease

Postnatal inactivation of Twinkle in neurons versus astrocytes led to grossly different phenotypes. TwKO^astro^ mice were significantly smaller in size than their control littermates already at the age of 3 months, and showed kyphosis and progressive weight loss (Fig. 1d, e), which was not due to reduced food intake (Supplementary Fig. 2a). At 5–6 months of age, the mice showed an abnormal symmetric hopping gait with compromised motor performance and weakness of hind limbs, with low pelvic posture, progressing to paraparesis (Fig. 1f, g). The animals had a limited lifespan of 7–8 months of age (Fig. 1h). The TwKO^neuro^ mice showed no difference in body weight from littermates until ~8 months of age (Fig. 1e). They developed moderate kyphosis, but showed no motor defects and appeared wild-type-like, until rapid deterioration of general condition and weight loss a few days prior to death at the age of 7–9 months (Fig. 1d–h). The overall gross morphology and size of the TwKO^astro^ and TwKO^neuro^ brains was unremarkable (Fig. 1i). No specific pathology was found in spinal cord (Supplementary Fig. 3).

**Fig. 2:**
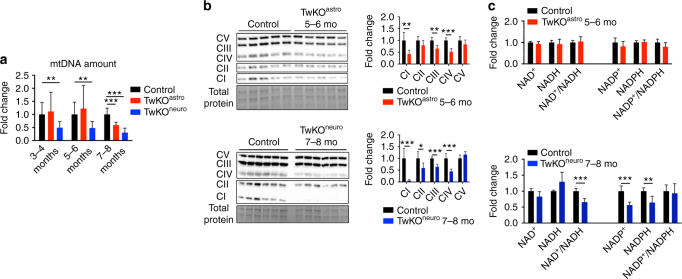
Mitochondrial consequences of Twinkle inactivation. **a** MtDNA amount in different ages in TwKO mice. **b** Western blot of OXPHOS complexes CI–CV and quantification of signals normalized to total protein. **c** NAD^+^, NADH, NADP^+^, NADPH amounts and their ratios in cortex lysates. The data are presented as mean and error bars indicate standard deviation. Statistical significance: *p < 0.05, **p < 0.01, ***p < 0.001

TwKO^neuro^, although asymptomatic at the age of 3–4 months, did demonstrate mtDNA depletion already at this age (Fig. 2a). TwKO^astro^ manifested with body weight loss and brain pathology starting from the age of 2–3 months, but showed significant mtDNA depletion only prior to death at 8 months (Fig. 2a). To assess consequences of the two genetic manipulations, we chose to analyze TwKO^astro^ mainly at 5–6 months of age, when the mice were already in advanced neurological disease, and neuronal-KO at 7–8 months, as only then they showed morphological findings and manifested with symptoms, and consequences to other cell types and brain pathology could be studied.

At terminal stage of TwKO^neuro^ and TwKO^astro^, mtDNA amount showed reduction to 25 and 50% of controls’ mean, respectively (Fig. 2a). Consequently, in TwKO^astro^, mtDNA-encoded RC enzyme complex amounts (CI, III, IV) were reduced in the brain tissue, with also OXPHOS enzyme activities, measured by high-resolution respirometry (Fig. 2b; Supplementary Fig. 2b). Fully nuclear-encoded CII showed normal protein amount. TwKO^neuro^ demonstrated a combined RC protein deficiency, with CI and CIV affected the most (~5% and 40% of the controls’ mean, respectively; CI contains seven mtDNA-encoded subunits; CIII one, CIV three and CV one; Fig. 2b). The severe CI deficiency in TwKO^neuro^ reflects the major contribution of neuronal mtDNA to total CI amount in mouse brain. In brain of TwKO^neuro^ mice also the overall redox status was altered with significantly reduced NAD/NADH ratio, contributed by CI deficiency, as the complex is a major acceptor of reducing equivalents from NADH. NAD is the precursor of both NADPH and NADP, and their levels were significantly decreased (Fig. 2c). As NAD and NADP form regulate major biosynthetic reactions in all cells, the effects of mtDNA depletion extend beyond RC, to the whole cellular metabolism.

### Loss of mtDNA maintenance activates astrocytes

**Fig. 3:**
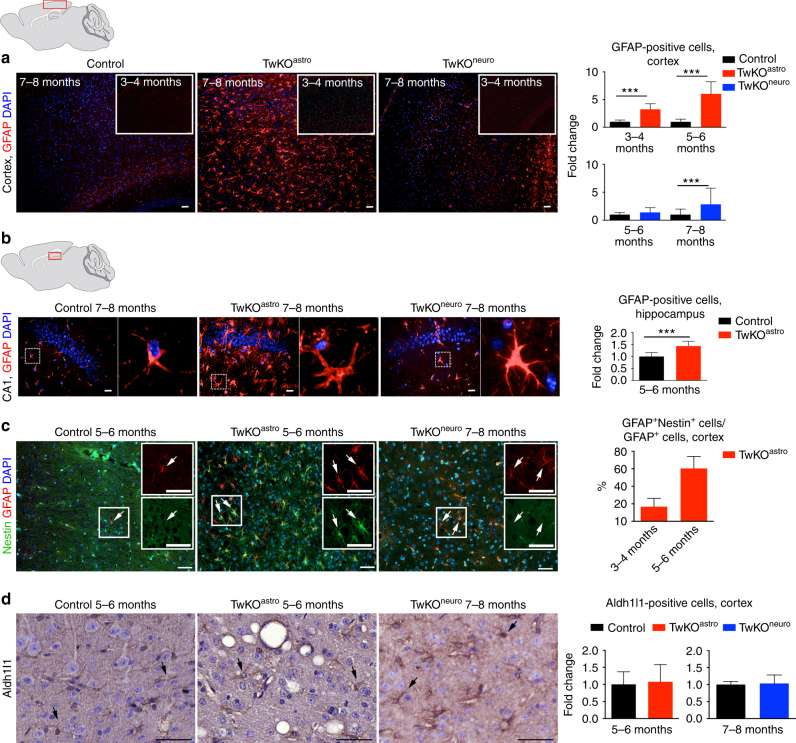
MtDNA loss in astrocytes causes massive astrocyte activation. **a**, **b** Astrocytes (GFAP, red) in somatosensory cortex (**a**) and in hippocampus, CA1 area (**b**) of 7–8-month-old mice and 3–4 months as shown in inset of (**a**). In **b**, boxed area is enlarged on right. **c** Astrocytes (GFAP, red) immuno-co-stained with reactive astrocyte marker (Nestin, green). Insets enlarge the boxed area. Arrows show the same cells. **d** Astrocytic marker Aldh1l1 immunohistochemistry, counterstaining hematoxylin. TwKO^astro^ show intensive vacuolization. Brain graphs show sampling site. Fluorescence figures are counterstained with DAPI (blue). Bar graphs on right hand side: corresponding quantifications of GFAP^+^ cells (cortex and hippocampus); GFAP^+^Nestin^+^/GFAP^+^, and Aldh1l1-positive cells in somatosensory cortex. The data are presented as mean and error bars indicate standard deviation. Statistical significance: *p < 0.05, **p < 0.01, ***p < 0.001. Scale bars: 20 μm (**a**), 50 μm (**b**–**d**)

Embryonic lethality of the full TwKO predicted progressive death of the targeted neurons or astrocytes. Unexpectedly, however, mtDNA depletion in astrocytes caused a remarkable increase of GFAP-positive cells with reactive astrocyte morphology in the targeted brain regions (Fig. 3a, b). Such morphology is typical for disease-related reactive gliosis. Activation was progressive, starting at 2–3 months of age (absent at the age of 1 month, Supplementary Fig. 4b), peaking in the cortex at terminal stage. The TwKO^neuro^ showed only modestly increased cortical astrogliosis at the terminal stage, with increased number of GFAP-positive cells of stellar morphology in cortex (Fig. 3a, b). Part of the population of reactive astrocytes in TwKO^astro^ also co-expressed nestin, another marker of reactive gliosis. Nestin-positive cells are not found in the cortex of controls or TwKO^neuro^ (Fig. 3c). The amount of GFAP^+^Nestin^+^ double-positive cells was progressive (<30% at the age of 3–4 months, and > 50% at 5–6 months). We then addressed the question, whether the remarkable number of activated astrocytes was due to their proliferation. In all three genotypes we found rare KI67-positive cells, marking cell division, but not co-localizing in the nuclei of the GFAP-positive cells (Supplementary Fig. 4c). Furthermore, most of the astrocytes in the mouse cortex are Aldhl1-positive, and in TwKO^astro^ they showed slightly swollen morphology, but their number was not changed (Fig. 3d). These data imply that the gliosis is due to astrocytes activated on site, but not their proliferation.

**Fig. 4:**
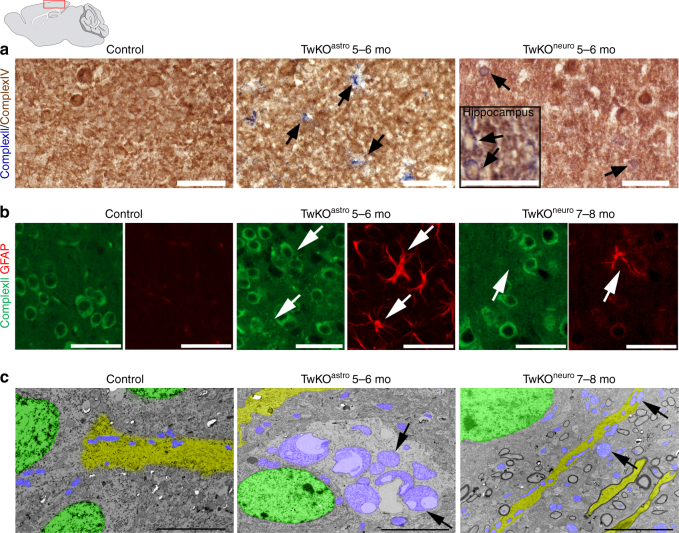
OXPHOS activity and mitochondrial structure in Twinkle KOs. **a** In situ histochemical activity analysis of mitochondrial respiratory chain enzymes in cortex. Brown: Complex IV, contains mtDNA-encoded subunits. Blue: fully nuclear-encoded Complex II. Arrows: respiratory chain deficient cells with astrocyte (TwKO^astro^) or neuronal (TwKO^neuro^) morphology. Inset in TwKO^neuro^: hippocampus, COX-deficient cells pointed with arrows. **b** Mitochondrial Complex II (SDHA, green) immuno-co-stained with astrocytes (GFAP, red), arrows point the same cells. TwKO^astro^ astrocytes show punctate mitochondrial accumulations. **c** Ultrastructure of mitochondria (arrows and purple pseudocolor). Green = nuclei, yellow = neuronal projections. Brain graph shows sampling site. Scale bars: 50 μm (**a**, **b**), 5 μm (**c**)

To examine whether the activated astrocytes were RC deficient, we tested in situ the mitochondrial RC enzyme activities and mitochondrial morphology. Consistent with mtDNA depletion in astrocytes of TwKO^astro^ mice, we found a large number of cells with astrocyte-like stellar morphology showing typical signs of RC deficiency: succinate dehydrogenase-positive (SDH; CII, nuclear-encoded) and cytochrome c oxidase-negative (COX; CIV, partially encoded by mtDNA)^25^ (Fig. 4a, Supplementary Fig. 5a). Also in TwKO^neuro^ we found COX-negative cells of neuronal morphology at 5–6 months of age, consistent with neuronal mtDNA depletion (Fig. 4a, Supplementary Fig. 5a). The activated astrocytes in TwKO^astro^ harbored accumulations of mitochondria, a sign of mitochondrial dysfunction, both perinuclearly and in cell processes (Fig. 4b, Supplementary Fig. 5b). GFAP-positive cells of controls or the reactive astrocytes of TwKO^neuro^ mice showed no such accumulations (Fig. 4b, Supplementary Fig. 5b). These findings indicate that the targeted cell populations developed RC deficiency as a consequence of lack of mtDNA-encoded RC subunits. Furthermore, the mitochondrial accumulation in astrocytic processes, similar that has been previously found for example in Purkinje dendrites of Twinkle-mutant Deletor mice^25^, suggests dysfunctional trafficking or impaired turnover of the organelle. Electron microscopic examination of TwKO^astro^ cortex revealed clusters of giant vacuolated mitochondria in cells with astrocytic morphology (Fig. 4c). In TwKO^neuro^, the neuronal mitochondria appeared swollen, but showed no vacuolization (Fig. 4c). The findings strongly suggest that (1) astrocyte functions are dependent on mitochondrial metabolism; (2) astrocyte activation is a cell-autonomous consequence of Twinkle inactivation and consequent mtDNA loss; (3) astrocyte activation caused by primary mtDNA depletion is different from secondary activation after neuronal mtDNA depletion, further pointing the importance of mtDNA maintenance for correct astrocyte functioning.

### MtDNA loss in astrocytes causes secondary neurodegeneration

**Fig. 5:**
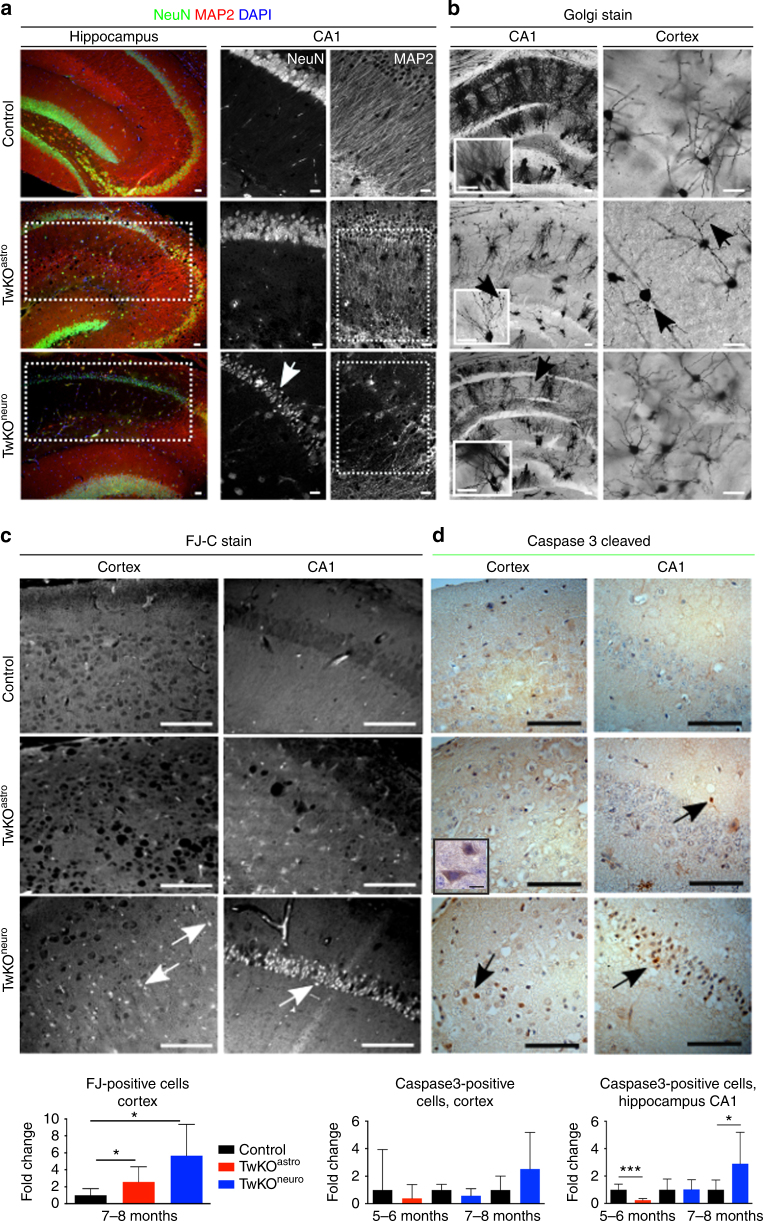
MtDNA loss in astrocytes or neurons challenges neuronal dendrite maintenance and causes neurodegeneration. **a** Immunofluorescent co-staining (overlay) of neuronal nuclei (NeuN, green), dendrites (microtubular MAP2, red) and nucleus (DAPI, blue) in hippocampus of 7–8 months old mice. **b** Neuronal fine morphology with Golgi staining in 7–8 months old mice. Arrows point gross morphological changes with dendritic degeneration in both KOs and varicosities in TwKO^astro^. **c** Dying neurons (arrows), Fluorojade-C (FJ-C) staining. Quantification below. **d** Apoptotic cells (arrows); immunohistochemical staining for cleaved caspase-3 and counterstaining with hematoxylin (parallel sections to FJ-C (**c**)). Quantification in somatosensory cortex and CA1 region of hippocampus below. Inset: enlarged caspase-3-positive cell. The data are presented as mean and error bars indicate standard deviation. Statistical significance: *p < 0.05, **p < 0.01, ***p < 0.001. Scale bars: 20 μm (**a**, **b**), 50 μm (**c**, **d**)

We then asked whether chronic astrocyte activation in TwKO^astro^ had secondary consequences for neurons. Twinkle inactivation in astrocytes resulted in significantly disorganized neuronal dendrite arborization in the hippocampal stratum radiatum, but the somata of pyramidal neurons appeared intact (Fig. 5a). To visualize the neuronal morphology in detail, we used Golgi staining of neurons. Indeed, dendrite complexity was dramatically decreased in both hippocampus and cortex of TwKO^astro^. The few and short dendrites carried dense varicosities (Fig. 5b), potentially as a consequence of deficient synaptic pruning. TwKO^astro^ secondarily challenged viability of cortical neurons close to terminal stage (Fig. 5c, d), but the death was not occurring through caspase-3-dependent manner. However, occasional caspase-positive neurons/glia were present in the neuropil of TwKO^astro^ mice (Fig. 5d). In the hippocampal CA1 region and cortex of TwKO^neuro^, Golgi-staining demonstrated short dendrites, decreased dendrite complexity and reduced neuronal body size, accompanied by abundant cell death, significantly more than in TwKO^astro^ (Fig. 5b–d). These results indicate that the early-onset phenotype of TwKO^astro^ mice is not explained by cell death. Instead, the chronic activation of the astrocytes challenges their functions in neuronal maintenance, leading to progressive changes in neuron structure and late-stage secondary neurodegeneration.

### TwKO^astro^ affects neuron subtypes and promotes inflammation

**Fig. 6:**
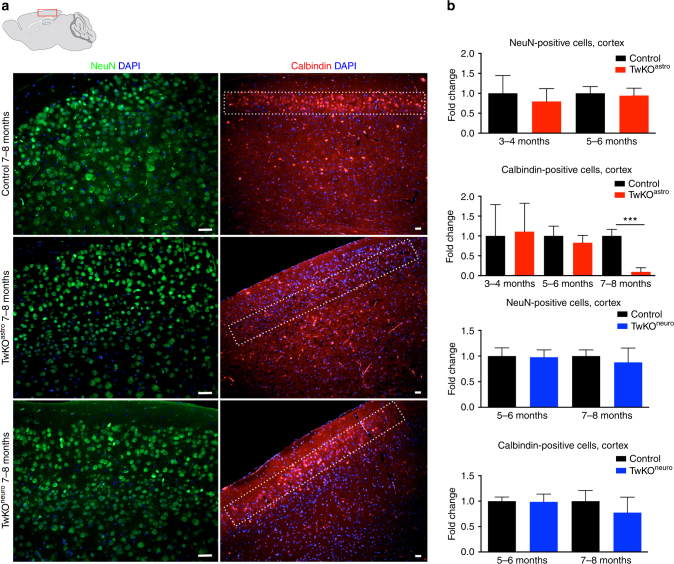
MtDNA loss in astrocytes depletes Calbindin-positive interneurons in cortex, upper neuronal layers. Immunofluorescnece staining of mid-sagittal sections of 7–8 months old mice, brain graph shows sampling site. **a** Neuronal nuclei (NeuN, green), Calbindin-positive neurons (red) and nucleus (DAPI, blue). **b** Quantification of NeuN-and Calbindin-positive neurons in somatosensory cortex, cortical layers I and II are boxed. Scale bars: 20 μm. The data are presented as mean and error bars indicate standard deviation. Statistical significance: ***p < 0.001

We then asked what types of cortical neurons were differentially affected by TwKO. The overall cellularity in the cortex was relatively well preserved in all mice (Fig. 6a). However, TwKO^astro^ mice showed a nearly complete loss of GABA-ergic calbindin-positive interneurons in the somatosensory cortical layers I-II at an advanced disease stage (Fig. 6a, b). In TwKO^neuro^ cortex, the calbindin-positive neurons remained relatively well preserved. As the cre-expression is prominent in these regions in both KO models, the TwKO^neuro^ data suggest relative resistance of these cortical neurons to mtDNA loss, but high sensitivity of these cells to dysfunction of their astrocytic support.

**Fig. 7:**
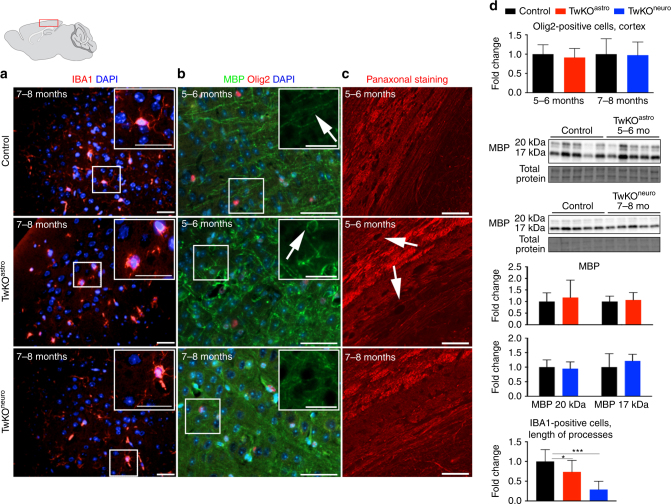
Inactivation of Twinkle in astrocytes leads to microglial activation and demyelination. Immunofluorescent staining of mid-sagittal sections from 6 months old mice, brain graph shows sampling site. **a** Immunofluorescent staining of microglia (IBA1, red) and nucleus (DAPI, blue) in cortex. **b** Myelin content (MBP, green), oligodendrocytes (Olig2, red), and nuclei (DAPI, blue). **c** Pan-axonal staining of long axons in corpus callosum. **d** Quantification of Olig2-positive cells and length of processes of IBA1-positive cells in cortex; western blot of MBP, and its quantification. Insets: enlarged boxed area. Scale bars: 20 μm (**a**), 50 μm (**b**, **c**). The data are presented as mean and error bars indicate standard deviation. Statistical significance: *p < 0.05, ***p < 0.001

Neuronal damage typically promotes an inflammatory response in the form of microglial activation. IBA1-positive microglia in both KO models adopted a rounded morphology, typical for activated microglia, whereas the control mice microglia had quiescent ramified morphology with elongated, branched processes (Fig. 7a). The microglial activation in TwKO^astro^ appeared earlier than in TwKO^neuro^, despite the moderate number of apoptotic cells in TwKO^astro^ compared to TwKO^neuro^, suggesting a link between astrocyte and microglial activation. Starting from the age of 5–6 months, the TwKO^astro^ mice had wide-spread chronic microglial and astrocyte activation accompanied by prominent myelin disorganization, while the number of Olig2-positive oligodendrocytes and total MBP content were unchanged (Fig. 7b, d). The axonal structure in the white matter of corpus callosum did not show signs of disruption (Fig. 7c). Myelin abnormalities were not present in TwKO^neuro^. These results indicate that chronic astrocyte activation and dysfunction promote inflammation and myelin changes.

### TwKO in astrocytes results in spongiotic encephalopathy

**Fig. 8:**
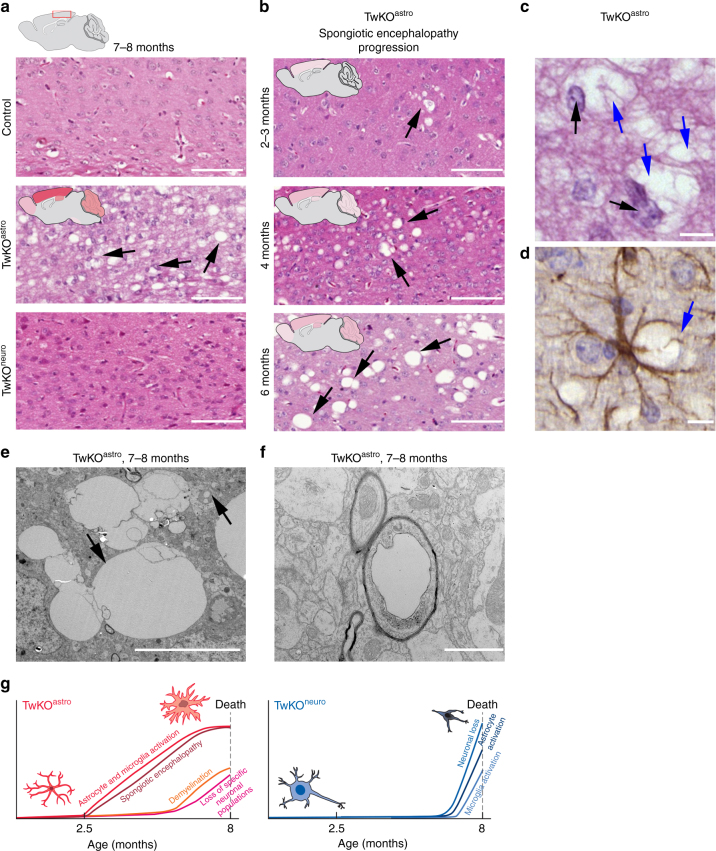
Inactivation of Twinkle in astrocytes leads to brain vacuolization and spongiotic encephalopathy. **a** Cortical morphology with hematoxylin/eosin staining from terminal stage of TwKO^astro^ and TwKO^neuro^ mice. Representative images of the brain mid-sagittal sections. Extensive brain vacuolization in TwKO^astro^ indicated with arrows. **b** Spongiotic encephalopathy of TwKO^astro^ has progressive nature. Brain graphs show the affected regions with severity of pathology demonstrated with intensity of the color shading. **c** Vacuoles (blue arrows) and astrocyte nuclei (black arrows) in hematoxylin/eosin staining. **d** Immunohistochemistry of GFAP in TwKO^astro^ and vacuoles enwrapped by an astrocyte (blue arrow). **e** Representative electron micrograph of vacuoles. Upper arrow indicates a vacuolated mitochondrion, lower arrow indicates group of vacuoles in the brain of TwKO^astro^. **f** Representative electron micrograph of vacuolated myelinated axon. Brain graph shows sampling site. Scale bars: 100 μm (**a**, **b**); 10 μm (**c**), 5 μm (**d**). **g** Time-course development of brain pathology in TwKO^astro^ and TwKO^neuro^ mice

The most remarkable pathological consequence of astrocyte-specific TwKO was development of spongiotic vacuolization of the brain (Fig. 8a). The vacuoles appeared in a progressive manner, starting from cortex at 2 months of age (not present at 1 month of age, Supplementary Fig. 4a). At the terminal stage, we found a severe spongiotic encephalopathy with profound vacuolization of the cortex and to a lesser extent of hippocampus, cerebellum, and olfactory bulb (Fig. 8b). The vacuoles are often formed close to intact astrocytic nuclei, and activated astrocytes enwrap the vacuoles (Fig. 8c, d). Ultrastructural analysis revealed clusters of round and oval shaped vacuoles of varying sizes, ranging from 1 to 15 μm (Fig. 8e). We also found vacuolization of myelinated axons at terminal stage, indicating neuronal degeneration (Fig. 8f). Intriguingly, these changes of TwKO^astro^ resemble closely the spongiotic pathology in infantile mitochondrial diseases, especially Leigh syndrome and Alpers–Huttenlocher syndrome, the latter caused by defects of mtDNA maintenance^3,6,9,26^.

Figure 8g summarizes the consequences of Twinkle inactivation in the two mouse models and the progression of different changes.

## Discussion

Glial cells constitute at least half of the cells in human neocortex^27^. They are a metabolically active and dynamic cell type, but considered to be mainly glycolytic, compared to high oxidative reliance of neurons. We report here that mtDNA maintenance is of absolute importance for astrocyte function, as Twinkle loss and progressive mtDNA depletion in these cells causes an early-onset, severe neurological disease, whereas a similar defect in neurons results in late-manifesting rapidly progressive disease course. The manifestations of TwKO^astro^ are among the most severe reported in any model targeting astrocytes in vivo^28,29^. Our findings are not consequences of a general decline of mitochondrial respiratory functions, because inactivation of COX assembly factor, COX10, does not lead to severe brain pathology^19^. Therefore, we claim that mtDNA helicase and replisome activity or mtDNA amount contribute to signaling that modulates astrocyte activity.

Surprisingly, mtDNA loss in TwKO^astro^ induced a wide-spread chronic activation of astrocytes, followed by severe secondary changes in neuronal morphology and progressive spongiotic encephalopathy, with relative sparing of neurons. The findings closely resemble those in children with mitochondrial encephalopathies, Alpers–Huttenlocher and Leigh syndromes. The neuropathologic characteristics of these disorders include spongiotic encephalopathy with astrocyte activation—often described as glial scar—relative sparing of neurons and cortical laminar necrosis^3,6,30,31^. Such pathology is also typical for adult-onset mitochondrial spinocerebellar ataxia-neuropathy-epilepsy syndromes caused by mtDNA maintenance defects^32,33^. We propose that chronic activation and dysfunction of astrocytes, as a consequence of mtDNA maintenance defect, is a primary underlying cause of spongiotic mitochondrial encephalopathies of children.

What type of dysfunction could underlie the spongiosis? Normal astrocytes promote CNS homeostatic functions by pruning synapses, maintaining axons, providing metabolites and trophic factors for neurons, and phagocytosing myelin debris^34^. However, during a CNS insult, activation of astrocytes may both promote and hinder recovery: microglial activation was reported to induce neurotoxic transformation of astrocytes, to “A1” type astrocytes. These cells lose important homeostatic functions, which leads to poor maintenance of synapses, neurodegeneration, and myelin changes^34–36^. The neurotoxic factor remains still unknown. We find activated astrocytes enwrapping pathological vacuoles, and neuronal morphology and myelin showing major secondary changes, indicative of possible toxicity. As increasing microgliosis in TwKO^astro^ follows astrocyte activation, it remains possible that the interaction of the two cell types contributes to pathology, with devastating secondary consequences to CNS.

Our evidence suggests that defective mtDNA maintenance in astrocytes drives their chronic activation, which promotes pathology of mitochondrial encephalopathy in mice. The findings offer an alternative mechanistic explanation for pathogenesis of the most common mitochondrial encephalopathies in infants. Furthermore, the results emphasize the potential importance of astrocyte dysfunction in all primary and secondary disorders with mtDNA maintenance defects, including dopaminergic neurons in Parkinson’s disease^37^. We propose that supporting of astrocytic mitochondrial function should be considered as an attractive target for intervention, to combat neurodegeneration.

## Methods

### Generation of knockout mice and phenotype testing

All the animal experiments were conducted according to relevant national and international guidelines, and approved by the Finnish Committee of Experimental Animal Research. The generation and validation of the loxP-mice is described elsewhere^38^. *Cre*-expressing mouse lines: [Tg(Pgk1-cre)1Lni, Camk2a-cre, GFAP(73.12)-cre and mT/mG; The Jackson Laboratory, Stock No: 2178050, 005359, 012886, 007576 ME, USA] were crossed to lox-P mice. *Cre*-mediated excision of the floxed fragment produced a frame-shift mutation leading to a premature stop codon. For staging, the day of vaginal plug was counted as embryonic day 0.5 (E0.5). For PGK-cre-mice, embryos were collected at E7.5, at the time of which all genotypes were present. The astro/neuro-TwKO offspring were born in Mendelian ratios. The mice were regularly followed up by inspection for activity, and weighted. Beam walk test, conducted as previously published^39^. Briefly, the mice were trained to navigate the 1 m poles of two different diameters (1 and 1.5 cm) for two constitutive days and recorded on the third day. We also used the grip strength tester (BioSEB, USA) with each mouse recoded three times with 5 min intervals.

### Tissue collection, expression analysis

The animals were sacrificed with CO_2_ and decapitated. One brain hemisphere was snap-frozen in liquid nitrogen and stored at −80 °C, and the other was sliced and immersion fixed in 4% PFA for immunohistological analysis. Cortex DNA and RNA isolation was performed using NucleoSpin TriPrep kit (Macherey-Nagel). The deletion caused by cre-excision was analyzed from *Twinkle* cDNA by PCR with the following primers sites 5′-CGTTTTGAGGACCTGCCTCT-3′, 5′-TTGGACACCTGCAGATACCG-3′. Primers used for *Twinkle* quantification by quantitative PCR were following: *Twinkle* 5′-ACGAGCAGCTCTCCTCTGA-3′, 5′-TGCTGTCTGCAGTTCCTTGT-3′ normalized to β-2 microglobulin level (primers: 5′-CATGGCTCGCTCGGTGACC-3′, 5′-AATGTGAGGCGGGTGGAACTG-3′).

### Histology, immunohistochemistry, and immunofluorescence

The animals were terminally anesthetized with Mebunat (Orion, Finland) and perfused intracardially with 4% paraformaldehyde in PBS or sacrificed with CO_2_. Brains were removed, post-fixed with 4% paraformaldehyde in PBS followed by embedding in paraffin. All histological and immunoassays were carried out on 7–10 μm sagittal sections. Immunohistochemical and immunofluorescence stainings were described previously^40^ and performed on a minimum of *n* = 3 of each genotype. COX/SDH activity analysis is described elsewhere^38^. Images were acquired with either Zeiss AxioImager epifluorescent microscope or Zeiss LSM 780 confocal microscope. Primary antibodies were rabbit Calbindin (Abcam), GFAP (Millipore), ALDH1L1 (Abcam), cleaved Caspase 3 (Cell Signaling Technology), IBA1 (Wako), Olig2 (Merck Millipore), KI67 (Merck Millipore); mouse Nestin (Merck Millipore), SDHA (CII) (Abcam), NeuN (Chemion), GAD67 (Merck Millipore); Neurofilament Marker pan axonal cocktail (BioLegend); chicken MAP2 (Thermo Scientific); rat MBP (Nordic BioSite). Golgi staining was processed using the FD Rapid GolgiStain Kit (FD Neuro Technologies). Fluoro-Jade C was from Millipore. Morphometrical analysis of coronal 80-μm-thick sections was described in ref. ^41^. For cell counting, images from mid-sagittal sections were taken from three to six mice per genotype.

### Electron microscopy

Ultrastructural analysis was performed as described elsewhere^42^. Briefly, tissue samples were fixed in phosphate buffer with 2.5% glutaraldehyde, post-fixed with 1% osmium tetroxide, dehydrated through ascending concentrations of ethanol and embedded in Epon-resin. Ultrathin sections of 60 nm were cut with Leica ultramicrotome and post stained with uranylacetate and lead citrate. Sections were imaged on a Phillips CM12 electron microscope operated at 80 kV.

### Western blot

Protein from frozen tissue was extracted in 150 mM NaCl 50 mM Tris buffer with 1% Triton-X, pH 7.6. Proteins were separated by SDS-PAGE using 4–20% gradient gels and transferred to PVDF membranes. Blots were blocked with 5% milk in Tris-buffered saline with Tween 20 (TBST) buffer for 1 h and antibodies were incubated at +4 °C overnight. Bio-Rad TGS stain-free technology was used for quantification of the total protein signal. Images were acquired with ChemiDoc Imager and quantified with ImageLab software. Original blots are shown in Supplementary Fig. 6.

### NAD^+^, NADH, NADP^+^, and NADPH measurement

Pyridine nucleotide concentrations in tissues were measured using cycling assay similar to previous publication^43^.

### High-resolution respirometry

Oxygen consumption rates were measured using a high-resolution oxygraph (OROBOROS instruments using a substrate-uncoupler-inhibitor protocol as described elsewhere^44^). Specific oxygen consumption rates are expressed as pmol/(s*mg). Briefly, half of 6 months old cerebellum was homogenized with 6–8 strokes in a 2 ml potter-Elvehjem in respiration buffer (0.5 mM EGTA, 3 mM MgCl_2_, 60 mM Lactobionic acid, 20 mM Taurine, 10 mM KH_2_PO_4_, 20 mM HEPES, 110 mM d-sucrose, 1% fat-free BSA). Oxygen consumption rates were performed in the presence of pyruvate-glutamate-malate and activities determined with +ADP (CI), +succinate (CI + CII), maximal uncoupled respiration by FCCP titration, and CIV by ascorbate + TMPD.

### MtDNA copy number analysis

Analysis of mtDNA amount was done by quantitative PCR, as described elsewhere^45^. Briefly, level of mtDNA (primers: 5′-AGGAGCCTGTTCTATAATCGATAAA-3′, 5′-GATGGCGGTATATAGGCCGAA-3′) was normalized to a nuclear-encoded single-copy gene RBM15 (primers: 5′-GGACAGTTTTCTTGGGCAAC-3′, 5′-AGTTTGGCCCTGTGAGACAT -3′).

### Statistical methods

For statistical significance analysis, we used unpaired Student’s *t* test or one-way ANOVA followed by Bonferroni tests (*P* ≤ 0.05).

### Data availability

Authors can confirm that all relevant data are included in the paper and/or its Supplementary Information files.

## Electronic supplementary material

Supplementary Information

Peer Review File
