# Supplementary material for: Loss of mtDNA activates astrocytes and leads to spongiotic encephalopathy
Source: Nat Commun. 2018 Jan 4;9:70. doi: 10.1038/s41467-017-01859-9 (PMC5754366; doi:10.1038/s41467-017-01859-9)
Supplement: Supplementary file 1 — Supplementary Information [file 41467_2017_1859_MOESM1_ESM.docx]

**Supplementary Figure 1**


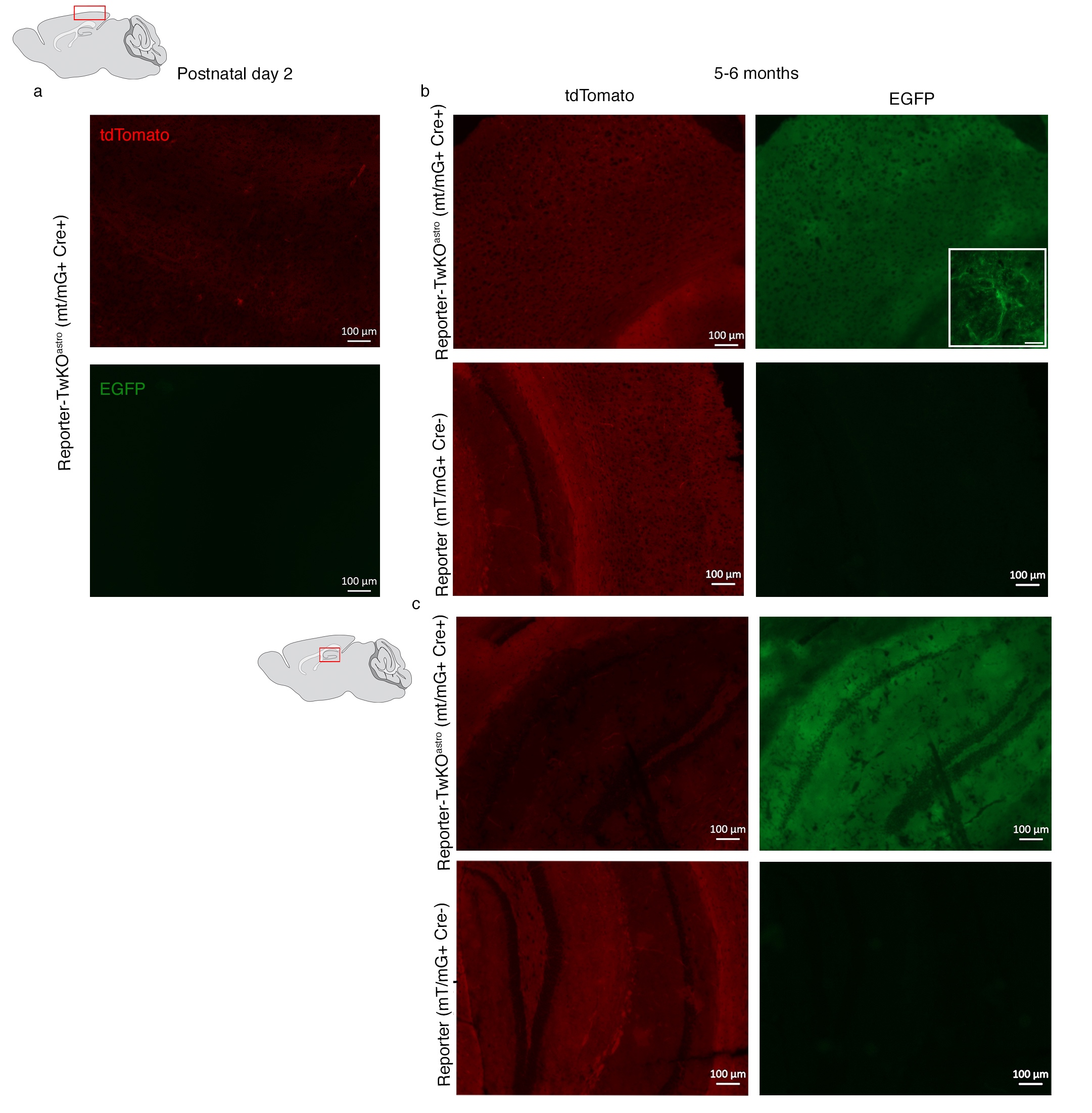


**Supplementary Figure 1**. **Gfap-cre transgene is expressed in the brain of TwKO^astro^.** Reporter mice express tdTomato under ubiquitous promotor, and start to express eGFP upon successful cre-recombination event. **(a)** Gfap-cre is not expressed at postnatal day 2, cortex shown **(b)** Representative images of somatosensory area of cortex and hippocampus **(c)** are shown, indicating widespread cre-activity in hippocampal and neocortical regions of TwKO**^astro^**. Inset: individual eGFP-expressing cell of astrocytic morphology. Brain graphs show sampling site. Scale bar 100 μm, inset: 20 μm.

**Supplementary Figure 2**


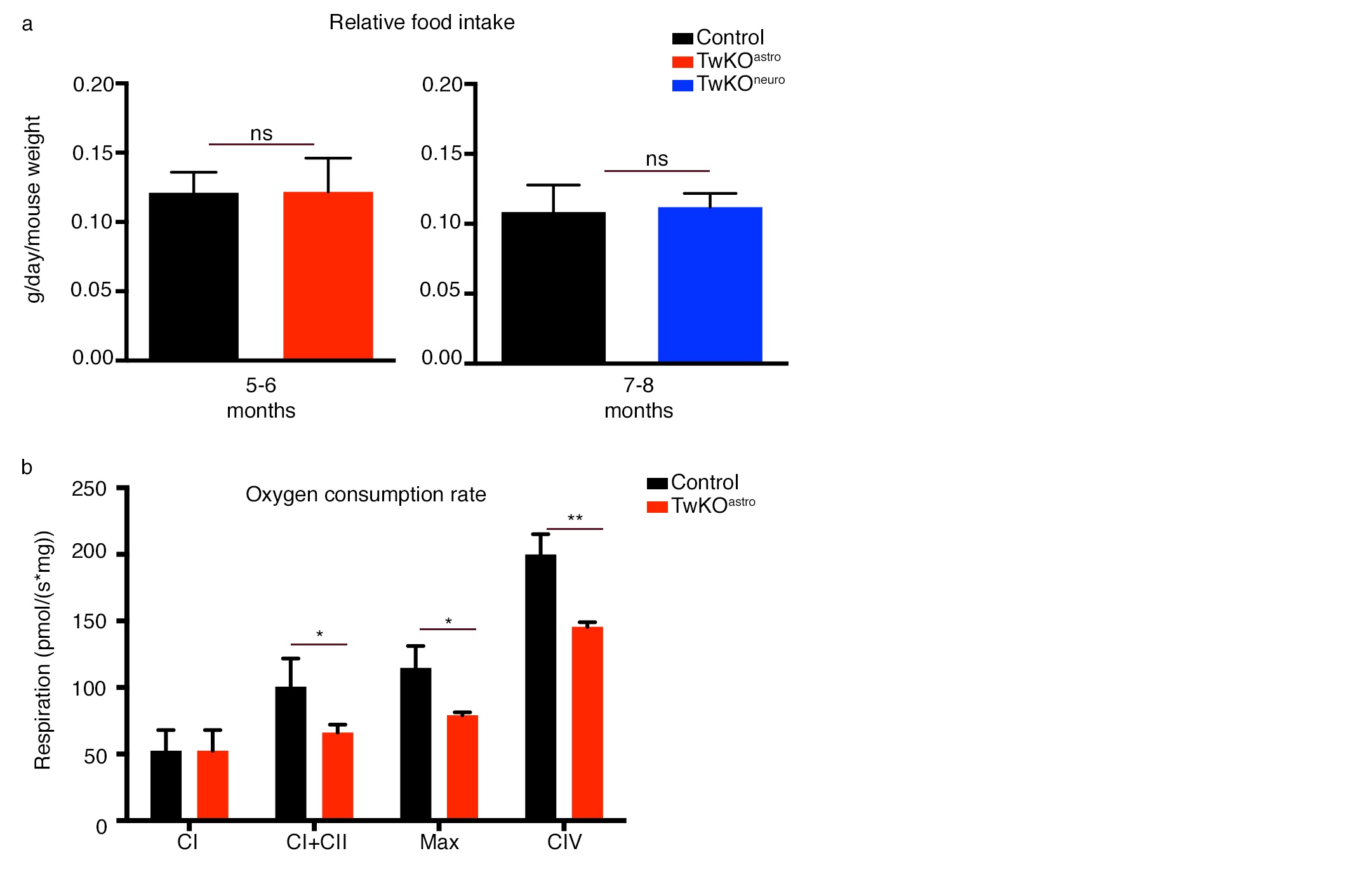


**Supplementary Figure 2**. **Food intake and oxygen consumption rate in knock out mice.** **(a)** Relative food intake normalized to body weight. **(b)** Oxygen consumption rate measured by oxygraph OROBOROS. The data are presented as mean and error bars indicate standard deviation. *P<0.05., **P<0.01

**Supplementary Figure 3**


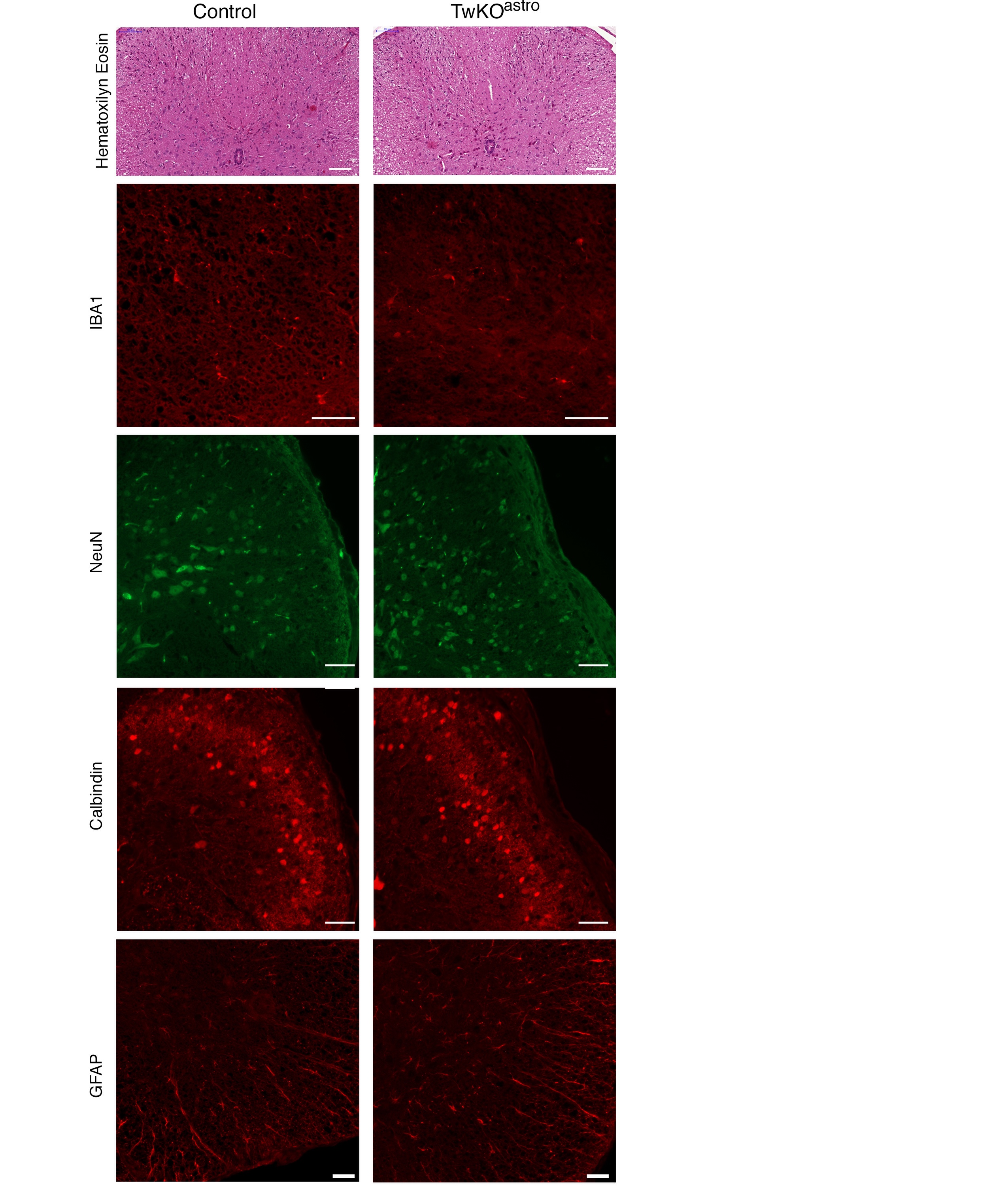


**Supplementary Figure 3**. **Spinal cord of TwKO^astro^ does not demonstrate pathology.** Hematoxylin/eosin and immunohistochemistry of spinal cord stained against IBA1, NeuN, Calbindin or GFAP are shown. Scale bar 100 μm (hematoxylin eosin), 50 μm (fluorescent pictures).

**Supplementary Figure 4**


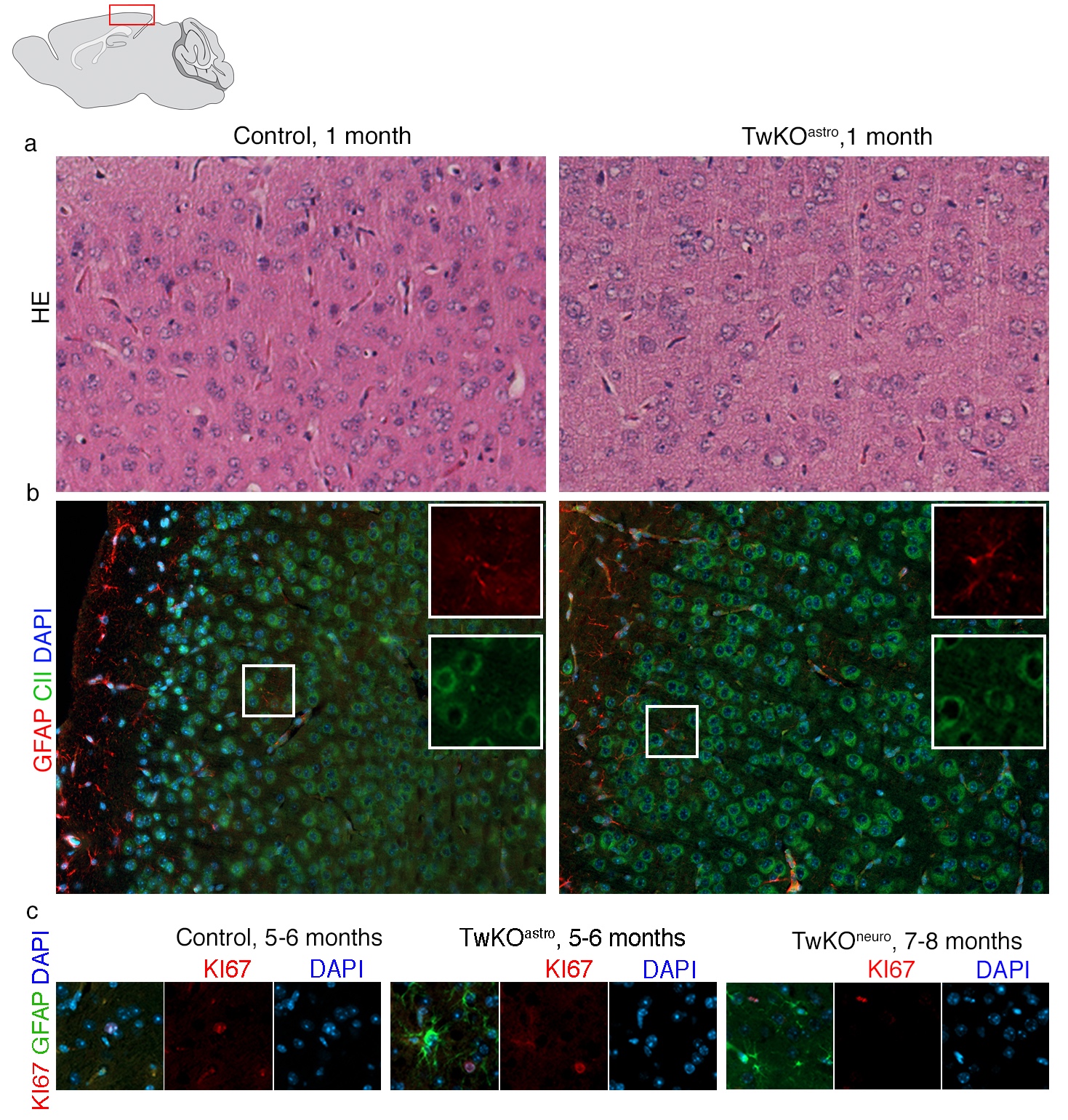


**Supplementary Figure 4**. **TwKO^astro^ brain does not demonstrate signs of brain pathology at 1 month of age. (a)** Representative picture of brain stained with hematoxylin-eosin **(b)** Fluorescent immuno-co-staining of mitochondrial complex II (SDHA) and astrocytic marker (GFAP). **(c)** Fluorescent immuno-co-staining of astrocytic marker (GFAP, green) and cell proliferation marker (KI67, red), nuclei (DAPI, blue). Brain graph shows sampling site.

**Supplementary Figure 5**


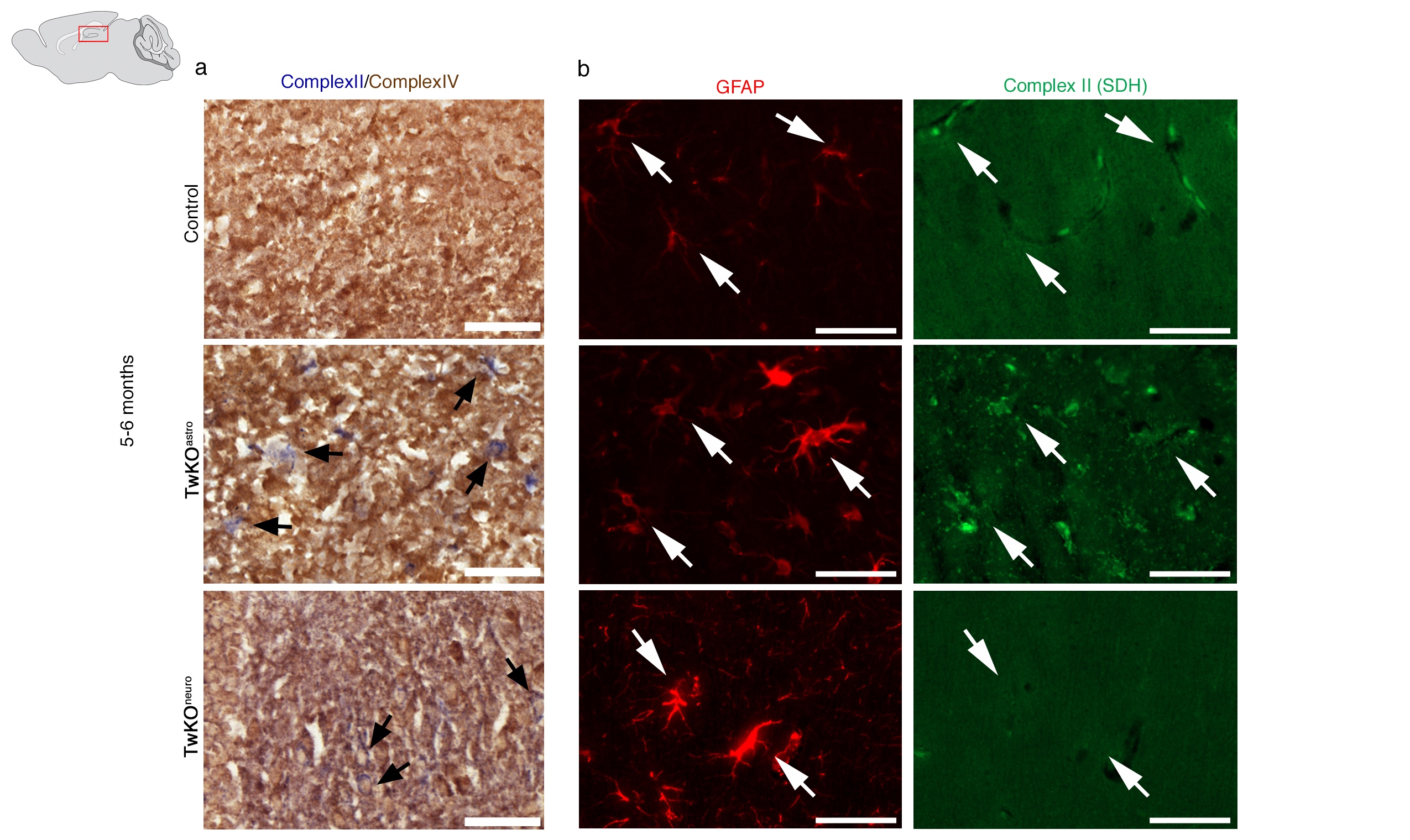


**Supplementary Figure 5**. **MtDNA loss in astrocytes causes massive astrocyte activation in hippocampus. (a)** *In situ* histochemical activity analysis of mitochondrial respiratory chain enzymes (Brown: Complex-IV, contains mtDNA-encoded subunits; blue: nuclear-encoded Complex-II). Arrows: respiratory chain deficient cells, astrocyte morphology. **(b)** Mitochondrial Complex-II (SDHA, green) immuno-co-stained with astrocytes (GFAP, red; arrows for the same cells). TwKO^astro^ astrocytes show punctate mitochondrial accumulations. Brain graph show sampling site. Scale bars 50 μm.


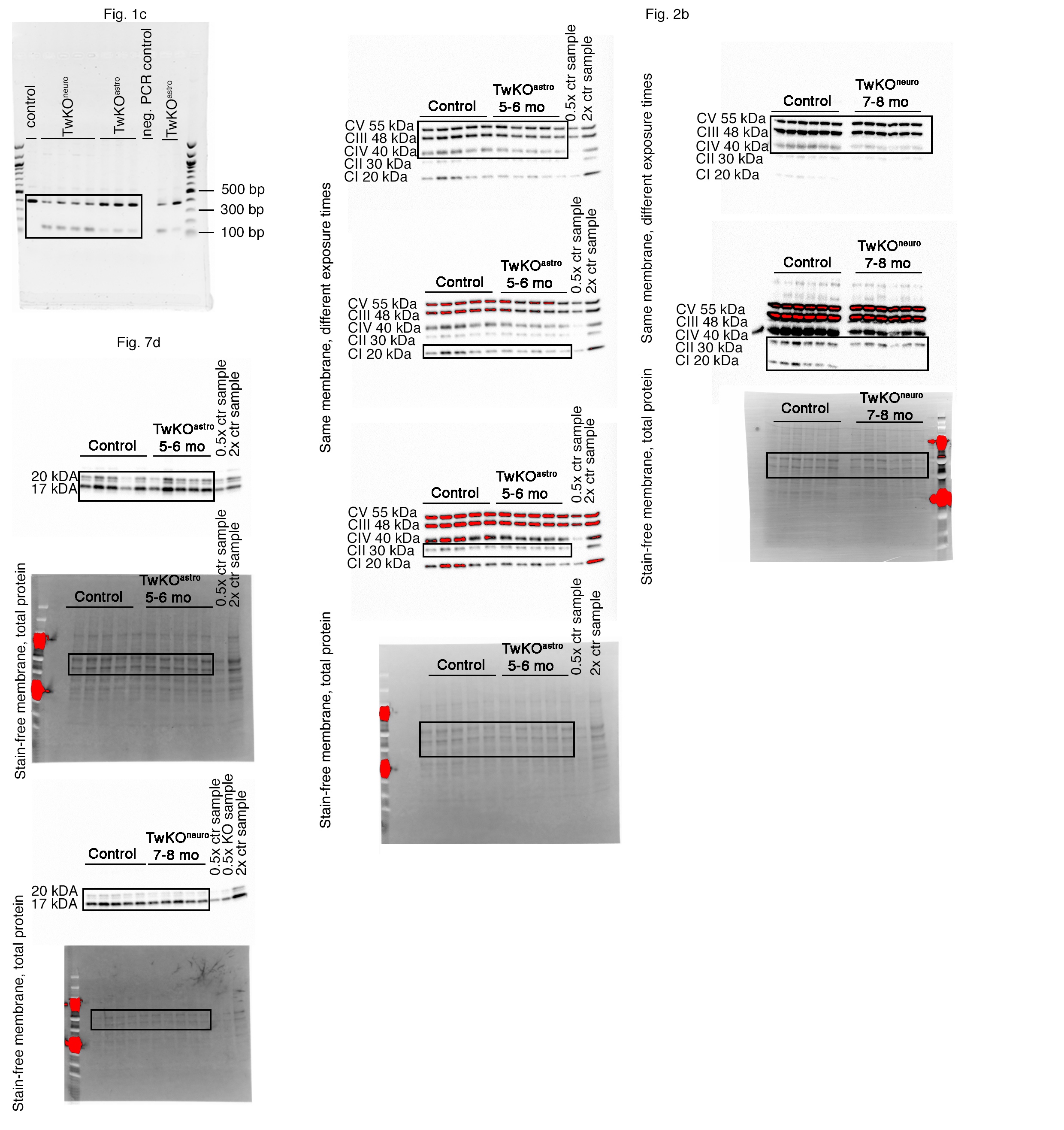


**Supplemetary Figure 6. Original blots and gels images of those included to the main text.** Portions of blots presented in the main paper are boxed.
