# Supplementary material for: Loss of mtDNA activates astrocytes and leads to spongiotic encephalopathy
Source: Nat Commun. 2018 Jan 4;9:70. doi: 10.1038/s41467-017-01859-9 (PMC5754366; doi:10.1038/s41467-017-01859-9)
Supplement: Supplementary file 2 — Peer Review File [file 41467_2017_1859_MOESM2_ESM.pdf]

Editorial Note: this manuscript has been previously reviewed at another journal that is not operating a transparent peer review scheme. This document only contains reviewer comments and rebuttal letters for versions considered at Nature Communications.

REVIEWERS' COMMENTS:

Reviewer #1 (Remarks to the Author):

The authors have addressed all the issues that I raised in the previous review cycle of this manuscript. I think this is an interesting and well performed study.

Reviewer #2 (Remarks to the Author):

The revised manuscript has addressed most of my concerns. With the additional experiments, graphs, and text changes, now the manuscript has become very interesting and exciting and I believe suitable for the publication in Nature Communications.

Reviewer #3 (Remarks to the Author):

The revised version of this paper has improved an already interesting work, which demonstrates the crucial role of astrocytes in the mitochondrial metabolism of the brain, using in vivo models. As the Authors emphasize in the Discussion, it seems that the presence of mtDNA itself, rather than an efficient bioenergetic supply, plays an essential role in regulating the behaviour and functions of astrocytes and their supportive action on neurons. This observation is interesting but also baffling, and the elucidation of the mechanism underpinning this phenomenon warrants further studies.
